# Synthesis and Use of [Cd(Detu)_2_(OOCCH_3_)_2_]*·*H_2_O as Single Molecule Precursor for Cds Nanoparticles

**DOI:** 10.1155/2013/907562

**Published:** 2013-10-31

**Authors:** Peter A. Ajibade

**Affiliations:** Department of Chemistry, University of Fort Hare, Private Bag X1314, Alice 5700, South Africa

## Abstract

Substituted thiourea ligands are of interest because they possess various donor sites for metal ions and their application in separation of metal ions and as antimicrobial agents. The coordination of the sulfur donor atom led to interest in them as precursor for semiconductor nanoparticles. In this study, cadmium(II) complex of diethylthiourea was synthesized and characterized by elemental analysis, FTIR, and X-ray crystallography. Single crystal X-ray structure of the complex showed that the octahedral geometry around the Cd ion consists of two molecules of diethylthiourea acting as monodentate ligands and two chelating acetate ions. The thermal decomposition of the compound showed that it decomposed to give CdS. The compound was thermolysed in hexadecylamine (HDA) to prepare HDA-capped CdS nanoparticles. The absorption spectrum showed blue shifts in its absorption band edges which clearly indicated quantum confinement effect, and the emission spectrum showed characteristic band edge luminescence. The broad diffraction peaks of the XRD pattern showed the materials to be of the nanometric size.

## 1. Introduction

Substituted thiourea shows more diverse coordination chemistry because of its conformational isomerism, steric effect, and the presence of donor sites on the substituted groups and intramolecular interactions [1–9]. Their chemistry has attracted attention due to various applications in separation of metal ions and as antimicrobial agents [2–8]. Coordination abilities of these ligands showed that the N-alkyl thiourea, with the coplanar N_2_CS skeletal atoms, can exist in two possible conformational forms, whereas three different conformations are possible for N,N′-dialkyl substituted thiourea [8]. Several studies have been done to understand the coordination chemistry of this type of ligand and its biological activities [7–10] and recently the use of the d^10^ metal complexes as precursors for metal sulfide nanoparticles [11–14]. Group 12 coordination compounds of thiourea have received attention in recent years for their nonlinear optical properties [9, 10] and their use as single source precursors for the preparation of semiconducting materials based on metal sulfide (MS) through thermal decomposition of the corresponding complexes [2, 11–15]. In this study, the synthesis, characterization, and thermal and single crystal X-ray studies of Cd(II) complex of diethylthiourea are presented. The complex was used to prepare hexadecylamine- (HDA-) capped CdxSy nanoparticles and the optical and structural properties of the nanoparticles determined.

## 2. Experimental Section 

### 2.1. Materials and Instrumentation

All the reagents and solvents used were of analytical grades and used without further purification. IR spectrum was obtained as KBr discs on a Perkin-Elmer Paragon 1000 FTIR spectrophotometer equipped with CsI window (4000–250 cm^−1^). Thermogravimetric analysis was performed on a Perkin Elmer thermogravimetric analyzer (TGA 7) fitted with a thermal analysis controller (TAC 7/DX). A flow of N_2_ was maintained with a heating rate of 10°C/min between ambient temperature and 800°C. 10–12 mg of the sample was loaded into an alumina cup, and weight changes were recorded as a function of temperature.

### 2.2. Synthesis of the [Cd(detu)_2_(OOCCH_3_)_2_]**·**H_2_O

The complex was prepared by addition of 2 mmol (0.533 g) of Cd (CH_3_COO)_2_·2H_2_O dissolved in 50 mL of absolute ethanol to a stirring 4 mmol of diethylthiourea (0.529 g) in 50 mL absolute ethanol. The mixture was refluxed for 6 h and filtered and left to evaporate slowly at room temperature. White crystals suitable for X-ray analysis were obtained after few days. Anal. Calc. For C_14_H_32_CdN_4_O_5_S_2_: C, 32.78; N, 10.92; H, 6.29, S, 12.50. Found: C, 33.01; N, 10.64; H, 6.03; S, 12.79%.

### 2.3. Single Crystal X-Ray Crystallography

Single crystals suitable for X-ray analysis for [Cd(detu)_2_(OOCCH_3_)_2_]·H_2_O were obtained after few days by slow evaporation of ethanolic solution of the complex. The data sets for the single crystal X-ray studies were collected with Mo K*α* radiation (k = 0.71073) at 100(2) K on a Bruker SMART APEX [16, 17] CCD diffractometer equipped with an Oxford Cryosystems low temperature device. Unit cell dimensions were obtained by least-squares refinement based on the setting angles of 925 reflections with theta (*h*) angles ranging from 2.50 to 26.50. The structure was solved by direct methods and refined by full-matrix least square on *F* ^2^ (SHELX97) [18, 19], and there is a water molecule in the asymmetric unit. All hydrogen atoms bonded to carbon were included in calculated positions. Those bonded to nitrogen and oxygen were found by the difference Fourier techniques and refined isotropically.

### 2.4. Synthesis of CdS Nanoparticles

About 0.5 g of [Cd(detu)_2_(CH_3_COO)_2_]·H_2_O was dissolved in 10 mL of tri^n^octylphosphine and injected into 6 g of hot hexadecylamine (HDA) at 150°C. A subsequent decrease in temperature of 20–30°C was observed. The solution was heated to 150°C and stabilized at this temperature for 1 h. The solution was then allowed to cool to 70°C, an excess of methanol was added, and a flocculants precipitate was formed. The solid was separated by centrifugation and redispersed in toluene. The toluene was removed under vacuum to give HDA-capped CdS nanoparticles. 

### 2.5. Characterization of Nanoparticles

XRD patterns were recorded by a Bruker D8 Advance X-ray diffractometer equipped with a proportional counter, using Ni-filtered Cu K*α* radiation (*λ* = 1.5405 A). Transmission electron microscopy (TEM) images were obtained on a Philips CM200 compustage transmission electron microscope with an accelerating voltage of 200 kV. The electronic spectra of the complexes were taken on a Perkin Elmer Lambda 250 UV-vis spectrometer. A Perkin Elmer LS 45 Fluorimeter was used to measure the photoluminescence of the nanoparticles. The SEM image was obtained in a Jeol, JSM-6390 LV apparatus, using an accelerating voltage between 15–20 kV at different magnifications. Composition and energy dispersive spectrum was processed using energy dispersive X-ray analysis (EDX) attached to the SEM with Noran System six software.

## 3. Results and Discussion

### 3.1. Molecular Structure of [Cd(detu)_2_(OOCCH_3_)_2_]·H_2_O

**Figure 1:**
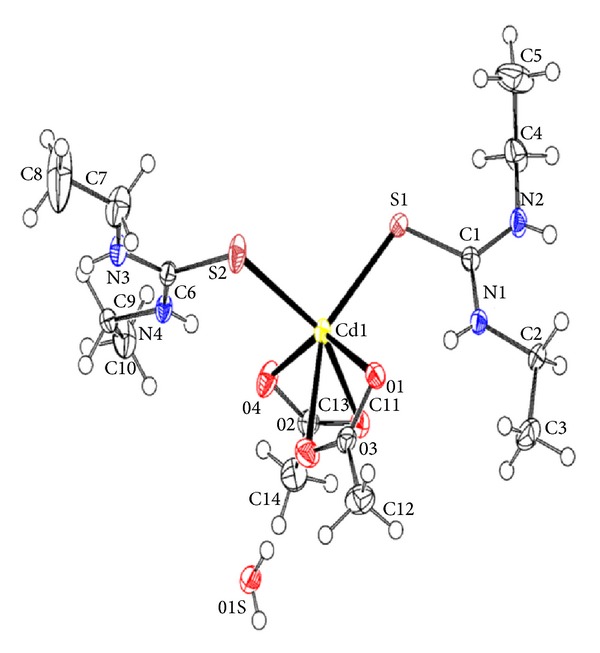
The molecular structure of [Cd(detu)_2_(CH_3_COO)_2_]·H_2_O showing the atom-labeling scheme. Displacement ellipsoids are drawn at 50% probability level. H atoms are represented by circles of arbitrary radius.

The molecular structure of the complex with atom numbering scheme is shown in Figure 1. The crystal data and structure refinement are presented in Table 1, and selected bond lengths and angles are given in Table 2. The cadmium in the molecular structure is a six-coordinate CdS_2_O_4_ form in which the Cd ion is coordinated to two monodentate diethylthioureas and two chelating acetate ions. The coordination polyhedral around Cd(II) is a distorted octahedron which might be due to the small bite angles of the acetate ions: O(2)–Cd(1)–O(1) [54.51(6)°] and O(4)–Cd(1)–O(3) [54.20(6)]. This also led to deviation of *trans *O(4)–Cd(1)–O(1), O(2)–Cd(1)–S(1) and O(3)–Cd(1)–S(2) bond angles significantly from 180° (ranges from 141.21(6)° to 151.48(4)°). The diethylthiourea ligands are *cis* to each other, and S–Cd(1)–S bond angle around the cadmium ion is 102.83(3)° which deviates considerably from 90°. This may be ascribed to the steric interaction between the substituents on the diethylthiourea. The C–S–Cd bond angles of 108.82(8)° and 111.59(8)° are slightly greater than the tetrahedral value.

**Figure 2:**
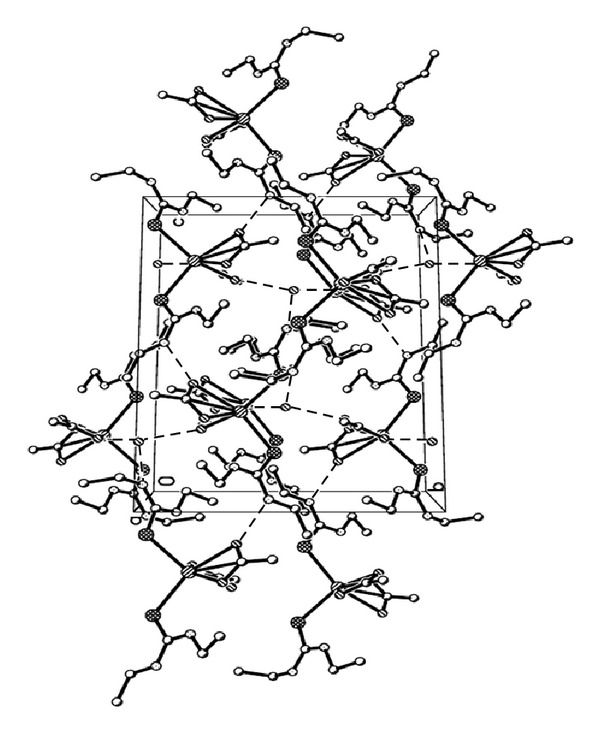
Crystal packing diagram of [Cd(detu)_2_(CH_3_COO)_2_]·H_2_O. Hydrogen bonds are indicated by dashed lines.

The S–C–N and N–C–N bond angles ranges from 118.51(17)° to 121.62(17)° and are within the expected range of tetrahedral. The C–N bonds (1.327(3) Å to 1.458(3) Å) and C–S bonds 1.727(2) Å to 1.730(2) Å are intermediate between single and double bonds. This may be attributed to the delocalization of electron in the thioamide bonds [20, 21]. The hydrogen bonding network within the molecule is between the acetate oxygen atom and NH and acetate oxygen atom and the hydrogen atoms of water within the crystal lattice (Table 3). Each water molecule within the crystal lattice is linked to three cadmium complex units via intermolecular hydrogen bonds. The other intermolecular hydrogen bonding is between the acetate oxygen atom and the NH of the diethylthiourea of neighbouring cadmium complex unit. The crystal packing (Figure 2) contained four cadmium complex units in two parallel chains. Each adjacent molecule is linked by intermolecular hydrogen bonds through a water molecule via the acetate oxygen atoms.

### 3.2. Infrared Spectra Studies

**Figure 3:**
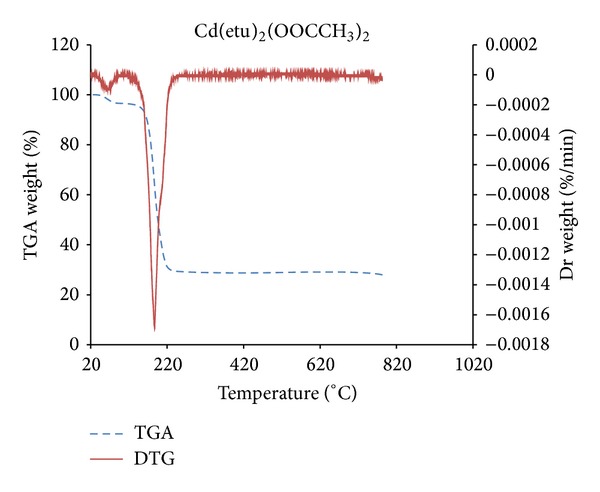
Superimposed TG/DTG curves of [Cd(detu)_2_(OOCCH_3_)_2_]·H_2_O.

The IR spectra of the ligand and complex were compared and assigned on careful comparison. The absorption band in the 3430–3200 cm^−1^ experienced only very slight changes in the complex. The N–H band occurs at 3275 cm^−1^ with a shoulder at 3430 cm^−1^ in the spectrum of the complex. The bands overlap with the C–H band to form a generally broad band. This is due to the presence of H-bonds networks in the molecular structure of the complex as confirmed by the single crystal X-ray structure of the compound (Figure 3). The *ν*(C=S) stretching vibrations occur at 747 cm^−1^, and the thioamide bond is observed as multiple band in the region 1554–1562 cm^−1^. This might be due to the overlap of the acetate ions with C–N bond stretching vibrations. The presence of multiple bands in the region 552–672 cm^−1^ can be attributed to the S–Cd–O interactions.

### 3.3. Thermogravimetric Studies

Thermogravimetric analysis of the compound, [Cd(detu)_2_(OOCCH_3_)_2_]·H_2_O, has been carried out to study the pyrolysis pattern in the temperature range 20–800°C. From the thermogram (TGA/DTG) in Figure 3, the complex undergoes two decomposition stages. The first decomposition occurs between 24°C and 78°C with a mass loss of 3.5%; and the second decomposition starts at 114°C and ends at 233°C with a 71% mass loss. This huge mass loss corresponds to the loss of the ligands and formation of CdS. The DTG peak temperature, for each of the decomposition stages are 61°C and 186°C, respectively. Considering the DTG peak temperatures, the first decomposition stage is due to entrapped solvent molecule. Thermal decomposition of related thiourea complexes reported in the literature has indicated that the residue formed corresponds to the formation of CdS [22]. Here, the weight of the residue calculated for CdS (3.68 mg) agrees favourably with the expected (3.65 mg). The DTA curve shows two pronounced endothermic peaks. The first and sharp peak is due to the melting of the complex at a temperature of 145°C. The second broad endothermic minimum which occurs at 182°C represents the decomposition of the complex. Comparing the TG curve with the DTA, it could be inferred that the second decomposition which gave CdS commenced in the liquid phase after the melting of the sample. 

### 3.4. Synthesis and Characterization of Nanoparticles

**Figure 4:**
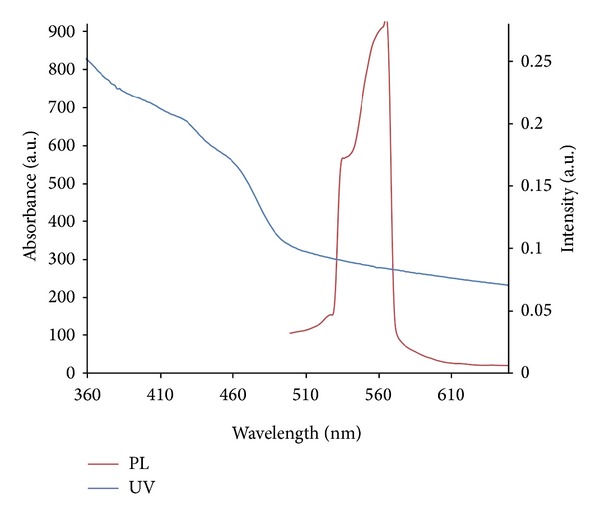
Absorption and photoluminescence spectra of the HDA-capped nanoparticles.

HDA-capped CdS nanoparticles were synthesized at 150°C using the cadmium complex. Optical properties of the nanoparticles were investigated by UV-vis and photoluminescence spectroscopy at room temperature (Figure 4) and show CdS nanoparticles with excitonic features at 420 nm and emission at 559 nm. The CdS nanoparticles showed quantum size effect which is manifested as a blue shift in their absorption band edges in comparison to that of the bulk. 

**Figure 5:**
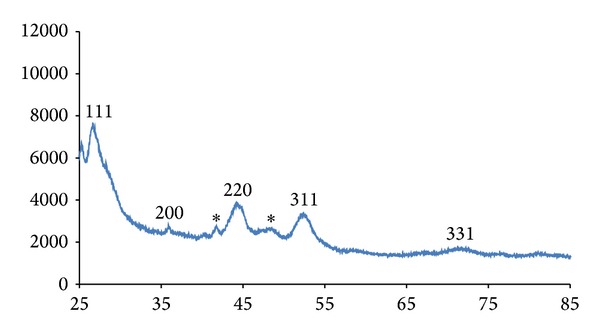
X-ray diffraction pattern of the CdS nanoparticles.

**Figure 6:**
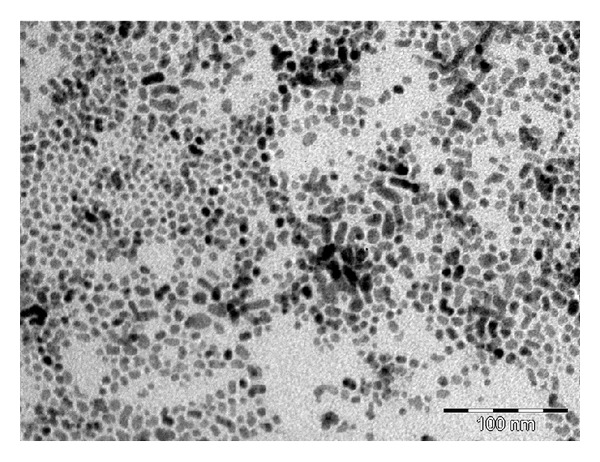
TEM image of the CdS nanoparticles.

**Figure 7:**
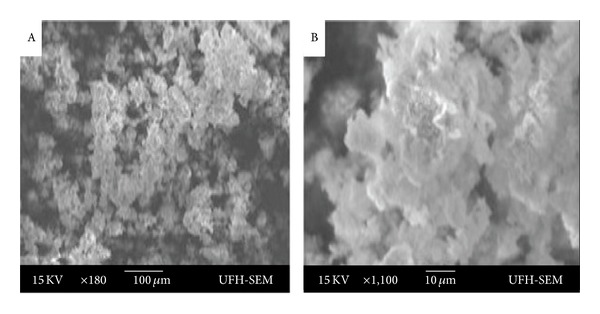
SEM Image of the nanoparticles.

The cadmium complex produced particles in the hexagonal phase with XRD patterns (Figure 5) indexed to 111, 200, 220, 311, and 331 for peaks with 2*θ* values of 26.4, 35.8, 43.9, 51.6, and 70.5, respectively. The peaks are generally broad, indicative of nanoscale particles. The TEM image of the CdS nanoparticles is presented in Figure 6. The nanoparticles are almost spherical in shape. A little aggregation is observed which could be ascribed to the effect of the small dimensions and high surface energy associated with nanodimensional particles. The sizes of the nanoparticles ranged between 5 and 19 nm. The SEM image of the prepared CdS nanoparticles is shown in Figure 7. The CdS nanoparticles have spherical morphology and show an average agglomerate size. The agglomeration of the nanoparticles may arise from their small dimension and high surface energy. 

## 4. Conclusions 

The reaction of cadmium acetate with diethylthiourea yielded the coordination complexes, [Cd(detu)_2_(OOCH_3_)_2_]·H_2_O. Recrystallization of the complex yielded well-defined crystals characterized by FTIR, elemental analysis, and single-crystal X-ray diffraction. Single crystal X-ray structure of the compound revealed that the coordination geometry around the Cd(II) is octahedron comprising of two diethylthiourea ligands and two acetate ions acting as bidentate chelating ligands. The single-source precursor route has been used for the preparation of CdS nanoparticles by thermolysis of the complex in hexadecylamine (HDA) to prepared HDA-capped nanoparticles. The absorption spectrum showed blue shifts in their absorption band edges which clearly indicated quantum confinement effect, and the emission spectrum showed characteristic band edge luminescence. The broad diffraction peaks of the XRD pattern showed the materials to be of the nanometric size with predominantly hexagonal phase. The TEM micrographs showed the CdS morphology to be almost spherical shape with particle sizes ranging between 5 and 19 nm.

## Figures and Tables

**Table 1:** Summary of crystal data and structure refinement for [Cd(detu)_2_(CH_3_COO)_2_]·H_2_O.

Compound	[Cd(detu)_2_(CH_3_COO)_2_]·H_2_O
Empirical formula	C_14_H_32_CdN_4_O_5_S_2_
Formula weight	512.96
Temperature	100(2) K
Wavelength	0.71073
Crystal system	Monoclinic
Space group	P2(1)/c
Unit cell dimensions	
*a* (Å)	11.755(4)
*b* (Å)	12.262(4)
*c* (Å)	16.609(5)
*β* (°)	108.071(5)
*γ* (°)	90
Volume (A^3^)	2275.9(12)
*Z*	4
*D*_calc_ Mg/m^3^	1.487
Absorption coefficient (mm^−1^)	1.172
*F*(000)	1056
Crystal size (mm)	0.25 × 0.20 × 0.20
Theta range (°)	1.82 to 26.43
Limiting indices	−14 ≤ *h* ≤ 14, −15 ≤ *k* ≤ 15, −20 ≤ 1 ≤ 20
Reflections collected	17814
Independent reflection	4672 [*R*(int⁡) = 0.0300]
Refinement method	Full-matrix least squares on *F* ^2^
Completeness to *θ* = 26.40	99.7
Data/restraints/parameters	4672/0/265
Goodness of fit on *F* ^2^	1.110
Final *R* indices [*I* > 2sigma(*I*)]	*R*1 = 0.0273, *wR*2 = 0.0607
*R* indices (all data)	*R*1 = 0.0324, *wR*2 = 0.0625
Largest diff. peak and hole e·Å^−3^	0.441 and −0.321

**Table 2:** Selected bond length and angles for [Cd(detu)_2_(CH_3_COO)_2_]·H_2_O.

Bond length (Å)		Bond angles (°)	
Cd(1)–O(4)	2.3077(18)	S(1)–Cd(1)–S(2)	102.83(3)
Cd(1)–O(2)	2.3714(18)	O(2)–Cd(1)–O(1)	54.51(6)
Cd(1)–O(1)	2.4200(18)	O(4)–Cd(1)–O(3)	54.20(6)
Cd(1)–O(3)	2.4753(17)	O(2)–Cd(1)–O(3)	79.28(6)
Cd(1)–S(1)	2.5587(8)	C(2)–S(1)–Cd(1)	108.82(8)
Cd(1)–S(2)	2.5656(9)	C(6)–S(2)–Cd(1)	115.59(8)
Cd(1)–C(13)	2.745(2)	O(4)–Cd(1)–S(1)	114.64
Cd(1)–C(11)	2.748(2)	O(2)–Cd(1)–S(1)	145.57(4)
O(1)–C(11)	1.271(3)	O(1)–Cd(1)–S(1)	95.20(4)
O(1)–C(13)	1.249(3)	O(3)–Cd(1)–S(1)	93.72(5)
O(1)–C(13)	1.252(3)	O(4)–Cd(1)–S(2)	97.49(4)
O(1)–C(13)	1.259(3)	O(2)–Cd(1)–S(2)	98.55(5)
S(1)–C(1)	1.727(2)	O(1)–Cd(1)–S(2)	99.43(4)
S(2)–C(6)	1.730(2)	O(3)–Cd(1)–S(2)	151.48(4)
N(1)–C(1)	1.327(3)	N(2)–C(1)–N(1)	119.1(2)
N(1)–C(2)	1.459(3)	N(4)–C(6)–N(3)	119.8(2)
N(2)–C(1)	1.324(3)	N(2)–C(1)–S(1)	120.10(17)
N(2)–C(4)	1.458(3)	N(1)–C(1)–S(1)	120.81(17)
		N(4)–C(6)–S(2)	121.62(17)
		N(3)–C(6)–S(2)	118.51(17)

**Table 3:** Hydrogen bond details for [Cd(detu)_2_(CH_3_COO)_2_]·H_2_O.

D–H*⋯*A	*d* (D–H)	*d* (H*⋯*A)	*d* (D*⋯*A)	<(DHA)
N(2)–H(2N)*⋯*O(1S)^#1^	0.77(2)	2.14(2)	2.886(3)	162 (2)
N(1)–H(1N)*⋯*O(1)	0.78(3)	2.09(3)	2.886(3)	174 (3)
O(1S)–H(1O)*⋯*O(2)	0.75(3)	2.10(3)	2.843(3)	170(3)
N(3)–H(3N)*⋯*O(3)^#2^	0.79(3)	2.05(3)	2.821(3)	166(3)
O(1S)–H(2O)*⋯*O(1)^#3^	0.78(3)	2.10(3)	2.882(3)	173
N(4)–H(4N)*⋯*O(4)	0.74(2)	2.05(3)	2.780(3)	170(3)

Symmetry transformations used to generate equivalent atoms: ^#1^ *x*, −*y* = 3/2, *z* − 1/2, ^#2^ *x*, −*y* + 3/2, *z* + 1/2, ^#3^−*x* + 1, *y* − 1/2, −*z* + 1/2.
